# Two-Step Derivatization of Amino Acids for Stable-Isotope Dilution GC–MS Analysis: Long-Term Stability of Methyl Ester-Pentafluoropropionic Derivatives in Toluene Extracts

**DOI:** 10.3390/molecules26061726

**Published:** 2021-03-19

**Authors:** Svetlana Baskal, Alexander Bollenbach, Dimitrios Tsikas

**Affiliations:** Institute of Toxicology, Core Unit Proteomics, Hannover Medical School, 30625 Hannover, Germany; baskal.svetlana@mh-hannover.de (S.B.); bollenbach.alex@gmail.com (A.B.)

**Keywords:** amino acids, derivatization, esterification, GC–MS, pentafluoropropionic anhydride, stability, toluene

## Abstract

Analysis of amino acids by gas chromatography-mass spectrometry (GC–MS) requires at least one derivatization step to enable solubility in GC–MS-compatible water-immiscible organic solvents such as toluene, to make them volatile to introduce into the gas chromatograph and thermally stable enough for separation in the GC column and introduction into the ion-source, and finally to increase their ionization by increasing their electronegativity using F-rich reagents. In this work we investigated the long-term stability of the methyl esters pentafluoropropionic (Me-PFP) derivatives of 21 urinary amino acids prepared by a two-step derivatization procedure and extraction by toluene. In situ prepared trideuteromethyl ester pentafluoropropionic derivatives were used as internal standards. GC–MS analysis (injection of 1 µL aliquots and quantification by selected-ion monitoring of specific mass fragments) was performed on days 1, 2, 8, and 15. Measured peak areas and calculated peak area ratios were used to evaluate the stability of the derivatives of endogenous amino acids and their internal standards, as well as the precision and the accuracy of the method. All analyses were performed under routine conditions. Me-PFP derivatives of endogenous amino acids and their stable-isotope labelled analogs were stable in toluene for 14 days. The peak area values of the derivatives of most amino acids and their internal standards were slightly higher on days 8 and 15 compared to days 1 and 2, yet the peak area ratio values of endogenous amino acids to their internal standards did not change. Our study indicates that Me-PFP derivatives of amino acids from human urine samples can easily be prepared, are stable at least for 14 days in the extraction solvent toluene, and allow for precise and accurate quantitative measurements by GC–MS using in situ prepared deuterium-labelled methyl ester as internal standard.

## 1. Introduction

**Scheme 1:**
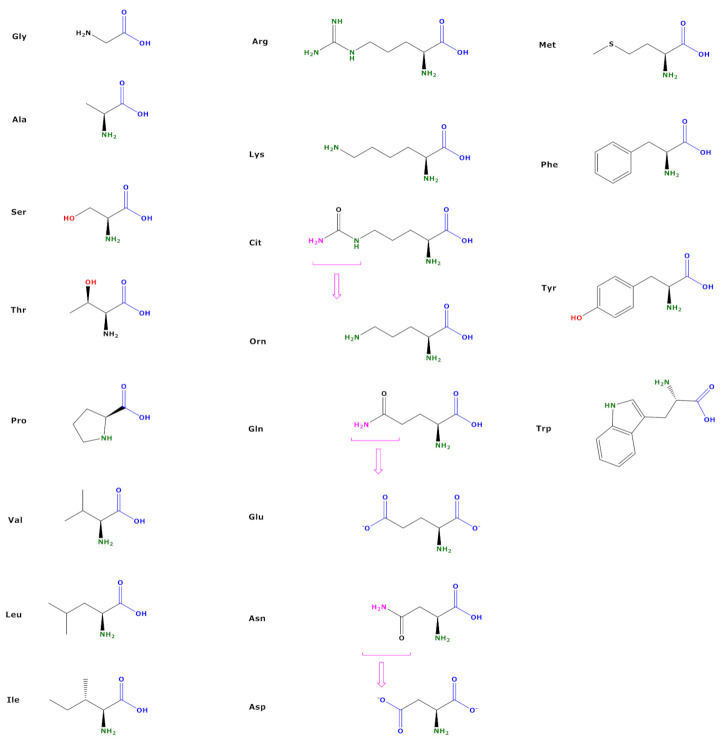
Chemical structures of the amino acids investigated in the present study. The functionalities being accessible to derivatization are colored. Blue, carboxylic group; Green, amine; Red, OH; Magenta, ureido, carbamoyl. Cit, citrulline; Orn, ornithine. The chemical structure of homoarginine (hArg), the methylene homolog of arginine (Arg) is not shown.

**Scheme 2:**
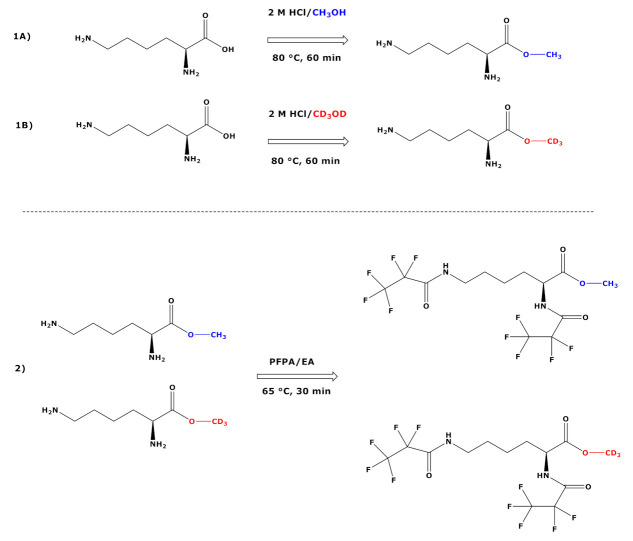
Two-step derivatization of lysine representative of the chemical class of amino acids. Separate esterification with 2 M HCl in CH_3_OH for biological amino acids (**1A**) and with 2 M HCl in CD_3_OD for synthetic amino acids (**1B**) to serve as internal standards. (**2**) Combined acylation with pentafluoropropionic anhydride (PFPA) in ethyl acetate (EA; 1:4, *v*/*v*).

Amino acids are carboxylic acids and contain at least one primary or secondary amine group and additional functionalities such as a hydroxyl (OH) group (Scheme 1). Amino acids are soluble in water and in water-miscible organic solvents such as methanol, but they are not soluble in water-immiscible organic solvents such as toluene. Amino acids and many of their metabolites are generally not accessible to gas chromatography (GC)-based analysis because they are not volatile and are thermally labile. Their injection in the gas chromatograph would lead to decomposition in the usually hot injector port to release small gases most likely including CO_2_, NH_3_ and H_2_O. Thus, native amino acids are not compatible with GC-based techniques such as gas chromatography-mass spectrometry (GC–MS). This trouble can be solved by chemical reactions of the carboxylic (COOH) groups, amine (NH_2_ and NH) groups, and OH groups with chemically reactive reagents to generate derivatives that are soluble in GC-compatible, water-immiscible organic, electro-neutral, volatile and thermally stabile solvents [1,2,3,4]. Biological samples contain amino acids and their metabolites. GC–MS analysis of all amino acids in biological samples such as blood and urine usually requires a two-step derivatization procedure to protect the above mentioned functionalities. Amino acids can be converted to their corresponding methyl esters by heating the samples for instance with 2 M HCl in methanol (CH_3_OH) or CD_3_OD [3] (Scheme 2, upper panel). This reaction is specific for carboxylic groups of amino acids. Thus, additional functionalities must be reacted with a second derivatizing reagent such as an organic anhydride (Scheme 2, lower panel) [2,5]. The reaction order of the two derivatization steps is of particular importance. Anhydrides of organic acids can react with all functional groups including carboxylic groups, but such derivatives are not stable enough [6].

In previous work [7,8], we found that a two-step derivatization step (Scheme 2) is useful for the reliable simultaneous quantitative measurement of amino acids by GC–MS in different biological samples including plasma, serum and urine. A major advantage of this derivatization procedure is the generation of stable-isotope labeled amino acids analogs from synthetic amino acids for use as the respective internal standards. The latter are synthesized in situ by performing the first derivatization step (60 min, 80 °C) in a solution of 2 M HCl in commercially available, isotopically highly pure tetradeutero-methanol (CD_3_OD) (Scheme 2) [5]. The fractions of unlabeled methyl esters (R–COOCH_3_) of biological amino acids and their deuterium-labeled methyl esters (R–COOCD_3_) are combined and subjected to the second common derivatization step (30 min, 65 °C) with pentafluoropropionic anhydride (PFPA) in ethyl acetate (1:4, *v*/*v*) (Scheme 2). Subsequently, organic solvents and excess PFPA are evaporated under a nitrogen stream, the residue is treated with borate buffer (200 µL) and immediately thereafter with toluene (200 µL). The sample is then vortex-mixed for 60 s to extract the amino acid derivatives into toluene, thereby keeping polar compounds such as pentafluoropropionic acid formed from hydrolyzed and reacted PFPA in the aqueous phase. A 150 µL aliquot of the upper toluene phase obtained by centrifugation (5 min, 800× *g*) is transferred into 0.2 mL microinserts which were placed in 1.5 mL autosampler glass vials, sealed tightly and subjected to GC–MS analysis [7,8].

Investigations of the stability of native analytes in their biological samples are essential in method validation. Stability studies of non-derivatized analytes in extracts such as in the mobile phase in liquid chromatography (LC)-based methods, for instance in autosampler vials during automated analysis, are widely performed and an indispensable part of method validation. Analogous stability studies in GC-based methods are relatively rare. Yet, this may be a particular concern for certain analytes such as amino acids derivatized with PFPA [6] and OH-rich carbohydrates derivatized with silylating reagents such as *N*,*O*-bis(trimethylsilyl)trifluoroacetamide (BSTFA) [9]. 

The aim of the present study was to investigate the long-term stability (14 days) in toluene extracts of the methyl ester pentafluoropropionyl derivatives (Scheme 2) of several urinary amino acids (Scheme 1). As the GC–MS analysis of histidine (His) and cysteine (Cys) by this method is not reliable enough [8], these amino acids were not investigated in the present study.

## 2. Materials and Methods

### 2.1. Chemicals and Materials

All synthetic amino acids were of analytical grade and were commercially obtained from various manufacturers (Sigma-Aldrich, Merck, Germany). Tetradeuterated methanol (CD_3_OD, 99% at ^2^H) and pentafluoropropionic anhydride were supplied by Aldrich (Steinheim, Germany). Methanol was obtained from Chemsolute (Renningen, Germany). Hydrochloric acid (37 wt%) was purchased from Baker (Deventer, The Netherlands). Ethyl acetate was obtained from Merck (Darmstadt, Germany). Glass ware for GC–MS (1.5 mL autosampler glass vials and 0.2 mL microvials) and the fused-silica capillary column Optima 17 (15 m × 0.25 mm I.D., 0.25 µm film thickness) were purchased from Macherey-Nagel (Düren, Germany). Separate stock solutions of amino acids were prepared by dissolving accurately weighed amounts of commercially available unlabelled and stable-isotope labelled amino acids in deionized water. Stock solutions were diluted with deionized water as appropriate.

### 2.2. Derivatization Procedures for Amino Acids in Human Urine Samples

A quality control human urine sample (#29) and six 24-h collected urine samples from six renal transplant recipients (#364, #367, #377, #382, #388, #390) of a previously reported study [10] were used. Urine aliquots (10 µL) were taken and evaporated to dryness under a stream of nitrogen. Methyl esters of the amino acids and their metabolites analyzed in the present study were prepared as follows (see Scheme 2). The residues of the urine samples were reconstituted in 100 µL aliquots of a 2 M HCl/MeOH solution and the vials were tightly sealed. Esterification was performed by heating the samples for 60 min at 80 °C. After cooling to room temperature, urine extracts were spiked with 10 µL aliquots of the newly synthesized trideutero-methyl esters, i.e., the internal standard mixture, in order to reach relevant concentrations with respect to human urine, and the solvents were evaporated to dryness under a stream of nitrogen. Aliquots (100 µL) of a daily prepared PFPA solution in ethyl acetate (PFPA-EA, 1:4, *v*/*v*) were added, the glass vials were tightly sealed and heated for 30 min at 65 °C to prepare *N*-pentafluoropropionic amides of the methyl esters. After cooling to room temperature, solvents and reagents were evaporated to dryness under a stream of nitrogen. Subsequently, residues were treated first with 200 µL aliquots of 400 mM borate buffer, pH 8.5, and immediately thereafter with 200 µL aliquots of toluene, followed by vortex-mixing solvent extraction for 60 s and centrifugation (4000× *g*, 5 min, 18 °C). Aliquots (150 µL) of the upper organic phase were transferred into autosampler glass vials equipped with 200 µL microinserts, the samples were sealed and subjected to GC–MS analysis. After each GC–MS analysis (i.e., injection of 1 µL toluene aliquots, quantification by selected-ion monitoring of specific mass fragments), the samples were sealed again and stored at room temperature until the next analysis. This procedure was repeated on days 2, 8, and 15. The peak areas measured and the concentrations calculated at various storage times were used to evaluate the stability of the derivatives as well as the precision and the accuracy of the method. The concentrations of the internal standards were within expected concentrations of endogenous urinary amino acids (Table 1).

### 2.3. Quantitative GC–MS Analyses of Amino Acids

All analyses were performed under routine conditions on a GC–MS apparatus consisting of a single-stage quadrupole mass spectrometer model ISQ, a Trace 1210 series gas chromatograph and an AS1310 autosampler from ThermoFisher (Dreieich, Germany) equipped with a 10 µL Hamilton needle. Toluene aliquots (1 µL) were injected in the splitless mode. The autosampler needle was cleaned automatically three times with toluene (5 µL) after each injection. Injector temperature was kept at 280 °C. Helium was used as the carrier gas at a constant flow rate of 1.0 mL/min. The oven temperature was held at 40 °C for 0.5 min and ramped to 210 °C at a rate of 15 °C/min and then to 320 °C at a rate of 35 °C/min. Interface and ion-source temperatures were set to 300 °C and 250 °C, respectively. Electron energy was 70 eV and electron current 50 µA. Methane was used as the reagent gas for negative-ion chemical ionization (NICI) at a constant flow rate of 2.4 mL/min. In quantitative analyses, the dwell time was 50 ms or 100 ms for each ion in the selected-ion monitoring (SIM) mode and the electron multiplier voltage was set to 1400 V.

The concentration of the amino in the human urine samples in quantitative analyses was determined in the SIM mode using one mass fragment (*m*/*z*) for the unlabelled amino acid and one ion for the corresponding mass fragment for the stable-isotope labelled amino acid serving as the internal standard (IS). The ions monitored are listed in Table 1. The peak area (PA) values of the urinary amino acids and of the respective internal standards were calculated automatically by the GC–MS software (Xcalibur and Quan Browser). The concentration of the amino acids was calculated by multiplying the peak area ratio of the endogenous urinary amino acid to the respective internal standard with the concentration of the respective internal standard. Statistical analyses and graphs were performed and prepared by GraphPad Prism 7 (San Diego, CA, USA).

## 3. Results and Discussion

The mean ratios of the PA values of the urinary amino acids to the PA values of the respective internal standards measured in the seven urine samples are summarized in Table 2. This Table indicates the inter-individual variation of the excretion of the analyzed amino acids. The concentrations of the amino acids in the 24 h collected urine samples of seven individuals varied from 18% to 20% for Met and from 89% to 93% hArg, indicating the biological variation of the urinary excretion of amino acids.

The coefficient of variation (CV) of the mean peak area ratio d_0_/d_3_ measured on days 1, 2, 8, 15 ranged between 0.47% for Orn/Cit and 12.9% for Gln/Glu. The higher CV values were due to higher values between days 1 and 2, and days 8 and 15 for some amino acids (Thr, Val) on the one hand, and lower values between days 1/2 and days 8/15 for other amino acids (Asn/Asp, Gln/Glu), one the other hand.

The mean peak area ratio values for the individual amino acids to their respective internal standards and the precision values (CV) in the toluene extracts of seven urine samples analyzed on days 1, 2, 8 and 15 are listed in Table S1. The CV values were below 10% for Ala, Thr, Gly, Ser, leu/Ile, Pro, Orn/Cit, Phe, Tyr, Lys, Arg, hArg and Trp. The CV values were in part between 10% and 28% for Val, Asn/Asp, Gln/Glu, Met. These data indicate that the Me-PFP derivatives of the analyzed amino acids are remarkably stable in the toluene extracts, with the stability apparently depending upon the amino acid and the biological matrix (i.e., urine).

**Figure 1:**
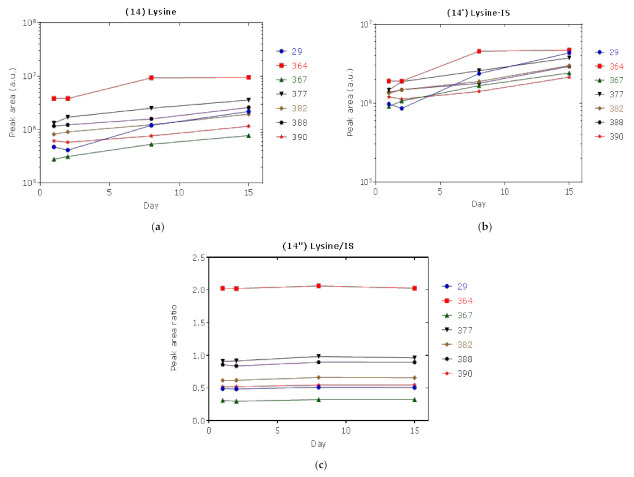
(**a**) Plots of the peak area of urinary lysine (upper left panel), (**b**) of the peak area of its internal standard (upper right panel), and (**c**) of the peak area ratio of lysine to its internal standard (lower panel) in seven 24 h collected urine samples against the storage time of the toluene extracts (day 1, 2, 8, 15). Note the decadic logarithm scale on the y axes in the left and middle panels. The plots of the other amino acids investigated in this study are found in the Supplementary Information. IS, internal standard; a.u., arbitrary units.

In order to investigate the stability of the Me-PFP derivatives of the endogenous amino acids and of the internal standards we plotted separately their peak area values against the storage time of the toluene extracts. The results of this analysis are illustrated in the Supplementary Figure S1 for all amino acids and in Figure 1 exemplarily for lysine.

On day 1 and day 2 very similar peak areas were measured in the respective urine samples for all amino acids (Figure S1, left panels) and their internal standards (Figure S1, middle panels). On day 8 and day 15 also very similar peak areas were observed for the respective urine samples for all amino acids and their internal standards, except that the peak area values were generally higher (Figure S1, left and middle panels). The volume of toluene phase did not decrease in the microinserts during the storage of the sample. Enrichment of the derivatives is therefore unlikely to explain the higher peak area values of the amino acid derivatives. Because the toluene phases were not vortex-mixed or shaken between the analyses (for practical reasons), it cannot be excluded that exceeding PFPA (ρ = 1.57 g/mL) and Me-PFP derivatives “precipitated” in the toluene phase. Yet, the peak area values of the endogenous amino acids and their internal standards increased by almost the same factor, finally resulting in remarkably constant peak area ratios practically in all urine samples (Figure S1, right panels). These observations suggest that the methyl ester-pentafluoropropionyl derivatives of unlabeled amino acids of urinary origin and of the deuterium-labelled amino acids are stable for at least two weeks when stored in toluene at room temperature and analyzed by GC–MS in laboratory routine. These observations also provide convincing evidence that trideutero-methyl esters of amino acids undergo almost the same physicochemical reactions including chemical derivatization and perfectly correct for all possible changes during the whole analytical procedure including incomplete derivatization.

The derivatization conditions used in the present study, including reaction temperature and time, for the derivatization of the amino acids were found in previous studies to be optimum [7,8]. Reaction of amino acids with methanol in 2 M HCl at 80 °C requires about 60 min for a mean yield of 85% for endogenous and synthetic mono- and dicarboxylic amino acids. Ureido and carbamoyl amino acids are unstable under the esterification conditions (Scheme 1) [6,8]. Citrulline (Cit) is hydrolyzed to form ornithine (Orn); asparagine (Asn) and glutamine (Gln) are also hydrolyzed to form aspartate (Asp) and glutamate (Glu), respectively; Orn, Asp and Glu are further esterified to form their dimethyl esters [8]. OH-groups containing amino acids (Thr, Ser, Tyr) are not affected by the HCl-catalyzed esterification.

After esterification, reaction mixtures must be evaporated to complete dryness in order to avoid hydrolysis and to increase the yield of the second consecutive derivatization with PFPA in EA. This reaction requires lower reaction temperature and shorter reaction time given the high reactivity of PFPA. The reaction solutions (PFPA-EA, 1:4, *v*/*v*) should be freshly prepared for maximum yield. As PFPA is highly reactive and non-selective, it reacts with many functional groups including primary and secondary amines to form *N*-pentafluoropropionyl derivatives, hydroxyl groups to form *O*-pentafluoropropionic esters, and carboxylic groups to form mixed anhydrides. While pentafluoropropionamides are resistant towards water, mixed anhydrides are not stable even in virtually anhydrous toluene [6]. Thr and Ser contain each one OH group which also react with PFPA to finally produce the methyl ester-*N*,*O*-pentafluoropropionic derivatives. The results of the present study suggest that the methyl ester-*N*,*O*-pentafluoropropionic derivatives of Thr and Ser are very stable in toluene. Tyr contains an aromatic OH group. Under the derivatization conditions used in our study (Scheme 2), the acidic OH group of Tyr is likely to react with PFPA, yet with the *O*-pentafluoropropionic ester being very easily and quickly hydrolyzing during the borate buffer/toluene extraction step. This has been previously demonstrated by trimethylsilylating the non-derivatized OH group of the Me-PFP derivative of Tyr with BSTFA at room temperature [11].

The reaction mixture of the second derivatization step contains numerous compounds of urinary origin including derivatized and non-derivatized amino acids, huge excess of non-reacted PFPA, as well as pentafluoropropionic acid from hydrolyzed and reacted PFPA. For optimum analysis and minimum contamination of the GC–MS apparatus, extraction of the Me-PFP derivatives is highly recommended. Water-immiscible and GC-compatible organic solvents are best suited. We selected toluene for solvent extraction because it is practically not miscible with water (solubility: 29 µM water in 1 L toluene; i.e., about 160 times less soluble than water in ethyl acetate) and has a lower density than water (ρ = 0.87 g/mL). These features facilitate phase separation and easy transfer of the upper organic phase into autosampler glass vials, thereby effectively avoiding water-carryover. The results of the present study underline the superiority of toluene for the extraction and long-term storage of Me-PFP derivatives of amino acids. Because of the very high molar excess of PFPA over amino acids and other substances of urine origin, it is likely that remaining PFPA is also extracted into toluene and serves as an “in injector” derivatization reagent like ethyl acetate in the analysis of nitrous acid [12].

Trimethylsilyl (TMS) derivatives of many amino acids in pyridine-methoxyamine-*N*-trimethylsilyl-*N*-methyltrifluoroacetamide (MSTFA) were found to degrade during the storage in glass vials in the autosampler within a few hours [13]. The stability increased considerably when the derivative solutions were stored at 4 °C (for 12 h), with maximum stability (for 72 h) when stored at −20 °C [13]. These results suggest that TMS derivatives of amino acids are not best suitable for GC–MS analysis.

Alkyl chloroformate is widely used for the GC–MS analysis of amino acids [2]. This derivatization procedure was automated for high-throughput analysis of different classes of substances including amino acids [14]. Yet, no stable-isotope labelled analogs were used for quantitation, with the mode of quantitation being not clearly described in that work. Stability studies of chloroform extracts dried over anhydrous sodium sulfate and at stored at −80 °C. The precision (RSD) of the GC–MS method was used as a measure of the derivatives stability. With respect to the derivatives of amino acids from human urine the stability was reported to be <20% for 6 days storage for the majority of amino acids. Phenylalanine, glutamate, cysteine have been excluded from stability analyses due to lacking linearity. 

## 4. Conclusions

Derivatization of the functional groups of amino acids, i.e., –COOH, –NH_2_, –NH, –OH, –SH first with 2 M HCl in CH_3_OH and then with PFPA in EA enables analysis of amino acids in biological samples such as human urine by GC–MS. Derivatization of the –COOH group(s) of synthetic amino acids with 2 M HCl in CD_3_OD from commercial sources is a useful approach to prepare stable-isotope labelled analogs for use as internal standards in quantitative analyses. Toluene is excellently suitable for the instantaneous extraction and long-term storage of urinary amino acids and their internal standards as methyl ester-pentafluoropropionyl derivatives. 

## Figures and Tables

**Table 1:** Summary of the GC–MS conditions used for the simultaneous quantitative determination of the indicated amino acids (AA) in human urine using their stable-isotope labelled analogs as internal standards (IS).

Amino Acid	Derivative	AA/IS (*m*/*z*)	Retention Time (min)	Dwell Time (ms)	IS (µM)
Ala	Me-PFP	229/232	3.73/3.70	100	220
Thr	Me-(PFP)_2_	259/262	4.07/4.05	50	165
Gly	Me-PFP	215/218	4.22/4.20	50	1100
Val	Me-PFP	257/260	4.44/4.42	50	33
Ser	Me-(PFP)_2_	207/210	4.46/4.43	50	330
Leu/Ile	Me-PFP	271/274	5.09/5.07	100	88
Asn/Asp	(Me)_2_-PFP	287/293	6.74/6.69	50	82.5
Pro	Me-PFP	255/258	7.18/7.16	100	22
Gln/Glu	(Me)_2_-PFP	301/307	7.93/7.89	100	275
Met	Me-PFP	289/292	7.94/7.92	100	55
Orn/Cit	Me-(PFP)_2_	418/421	8.60/8.58	50	27.5
Phe	Me-PFP	305/308	8.73/8.71	50	88
Tyr	Me-PFP	233/236	9.06/9.04	100	110
Lys	Me-(PFP)_2_	432/425	9.51/9.49	50	110
Arg	Me-(PFP)_3_	586/589	9.60/9.58	50	55
hArg	Me-(PFP)_3_	600/603	10.39/10.37	100	5.5
Trp	Me-(PFP)_2_	233/236	11.48/11.45	50	55

Abbreviations. AA, amino acid; IS, internal standard; *m*/*z*, mass-to-charge ratio; Me, methyl; PFP, pentafluoropropionyl. Orn, ornithine; Cit, citrulline; hArg, homoarginine.

**Table 2:** Mean peak area ratio and its coefficient of variation (CV) of the indicated endogenous amino acids (d_0_) in urine samples from seven volunteers to the respective internal standards (d_3_) as measured by GC–MS analysis of 1 µL aliquots of the toluene extracts stored at room temperature for several days.

Amino Acid	Mean Peak Area Ratio (d_0_/d_3_)				CV (%)	Inter-Indivual Variation
	Day 1	Day 2	Day 8	Day 15	All Days	CV (%)
Ala	0.427	0.417	0.413	0.415	1.49	74–79
Thr	0.653	0.666	0.588	0.584	6.87	30–32
Gly	0.538	0.535	0.550	0.561	2.18	41–44
Val	0.966	0.960	0.813	0.805	10.1	29–36
Ser	0.724	0.721	0.767	0.782	4.10	36–37
Leu/Ile	0.580	0.575	0.564	0.554	2.04	49–50
Asn/Asp	2.204	2.216	2.506	2.571	8.07	32–37
Pro	0.081	0.081	0.079	0.077	2.41	21–25
Gln/Glu	2.754	2.754	3.505	3.372	12.9	36–39
Met	1.526	1.531	1.889	1.869	11.9	18–20
Orn/Cit	1.091	1.096	1.103	1.100	0.47	34–35
Phe	0.381	0.338	0.350	0.348	5.25	64–67
Tyr	0.812	0.813	0.818	0.804	0.71	47–49
Lys	0.818	0.814	0.855	0.845	2.41	67–70
Arg	0.437	0.430	0.438	0.434	0.83	52–55
hArg ^a^	0.126	0.130	0.142	0.144	6.53	89–93
Trp	0.427	0.417	0.413	0.415	1.67	74–79

**^a^** hArg, homoarginine.

## Data Availability

The study did not report any data.

## Supplementary Materials

The following are available online, Table S1 Mean peak area ratio (coefficient of variation, CV) of endogenous amino acids in the seven human urine samples to the respective internal standard as measured by GC–MS analysis of 1 µL aliquots of the toluene extracts stored and room temperature for several days. Figure S1. Plots of the peak area of urinary amino acids (upper panel), of the peak area of their internal standards (middle panel), and of the peak area ratio of the amino acids to their internal standards (lower panel) in seven 24 h collected urine samples against the storage time of the toluene extracts (day 1, 2, 8, 15). Note the decadic logarithm scale on the y axes in the left and middle panels.

## Sample Availability

Not available.
