# Supplementary material for: Two-Step Derivatization of Amino Acids for Stable-Isotope Dilution GC–MS Analysis: Long-Term Stability of Methyl Ester-Pentafluoropropionic Derivatives in Toluene Extracts
Source: Molecules. 2021 Mar 19;26(6):1726. doi: 10.3390/molecules26061726 (PMC8003615; doi:10.3390/molecules26061726)
Supplement: Supplementary file 1 [file molecules-26-01726-s001.pdf]

Supplementary Materials

Two-Step Derivatization of Amino Acids for Stable-Isotope Dilution GC-MS Analysis: Long-Term Stability of Methyl Ester-Pentafluoropropionic Derivatives in Toluene Extracts

Svetlana Baskal, Alexander Bollenbach and Dimitrios Tsikas *

Institute of Toxicology, Core Unit Proteomics, Hannover Medical School; baskal.svetlana@mh-hannover.de (S.B.); bollenbach.alex@gmail.com (A.B.)
* Correspondence: Tsikas.dimitros@mh-hannover.de

Table S1. Mean peak area ratio (coefficient of variation, CV) of endogenous amino acids in the seven human urine samples to the respective internal standard as measured by GC-MS analysis of 1-μL aliquots of the toluene extracts stored and room temperature for several days.

Amino acid	Mean peak area ratio (CV, %)						
Urine No.	#29	#364	#367	#377	#382	#388	#390
Ala	0.306 (6.3)	1.10 (2.4)	0.14 (4.3)	0.45 (1.2)	0.28 (1.8)	0.407 (3.2)	0.242 (4.9)
Thr	0.692 (9.3)	0.884 (7.5)	0.292 (4.1)	0.727 (7.3)	0.718 (6.2)	0.568 (6.0)	0.469 (5.4)
Gly	0.56 (4.9)	0.84 (0.48)	0.18 (4.1)	0.78 (0.6)	0.426 (3.5)	0.64 (2.9)	0.39 (2.6)
Val	0.846 (12.6)	1.523 (12.9)	0.601 (4.9)	0.888 (12.6)	0.695 (10.7)	0.903 (9.8)	0.746 (5.3)
Ser	0.804 (4.3)	1.17 (3.1)	0.301 (3.3)	0.851 (6.9)	0.768 (3.4)	0.852 (3.7)	0.499 (5.3)
Leu/Ile	0.380 (1.8)	1.169 (1.7)	0.392 (2.6)	0.622 (5.6)	0.530 (1.1)	0.516 (1.8)	0.371 (3.8)
Asn/Asp	1.619 (5.1)	3.594 (6.2)	1.311 (28.3)	3.202 (11.1)	2.373 (6.2)	2.585 (6.2)	1.936 (6.5)
Pro	0.047 (3.6)	0.105 (4.9)	0.085 (4.5)	0.073 (2.4)	0.096 (2.8)	0.076 (3.9)	0.077 (1.3)
Gln/Glu	2.434 (6.8)	5.248 (10.3)	1.881 (13.2)	3.782 (12.6)	2.486 (16.0)	3.512 (19.7)	2.331 (14.5)
Met	1.881 (9.4)	2.053 (14.5)	1.255 (8.7)	2.005 (10.2)	1.445 (13.6)	1.800 (15.8)	1.486 (12.5)
Orn/Cit	0.858 (1.5)	1.791 (2.0)	0.555 (2.1)	1.288 (4.5)	1.038 (2.0)	1.049 (1.0)	1.104 (1.3)
Phe	0.317 (1.1)	0.861 (6.1)	0.159 (5.6)	0.354 (12.1)	0.262 (5.8)	0.304 (2.5)	0.225 (7.3)
Tyr	0.766 (1.6)	1.619 (1.5)	0.390 (1.5)	0.804 (3.3)	0.843 (0.7)	0.738 (2.7)	0.524 (1.0)
Lys	0.499 (2.49	2.034 (0.9)	0.314 (3.8)	0.943 (3.8)	0.639 (3.8)	0.872 (3.2)	0.533 (3.0)
Arg	0.263 (1.0)	0.934 (1.3)	0.418 (1.9)	0.316 (1.6)	0.477 (0.9)	0.315 (1.3)	0.321 (1.9)
hArg	0.100 (5.1)	0.408 (7.6)	0.055 (6.5)	0.068 (7.9)	0.151 (9.1)	0.120 (5.3)	0.049 (6.9)
Trp	0.306 (6.3)	1.102 (2.4)	0.141 (4.3)	0.454 (1.2)	0.277 (1.8)	0.407 (3.2)	0.242 (4.9)

Figure S1. Plots of the peak area of urinary amino acids (upper panel), of the peak area of their internal standards (middle panel), and of the peak area ratio of the amino acids to their internal standards (lower panel) in seven 24-h collected urine samples against the storage time of the toluene extracts (day 1, 2, 8, 15). Note the decadic logarithm scale on the y axes in the left and middle panels.

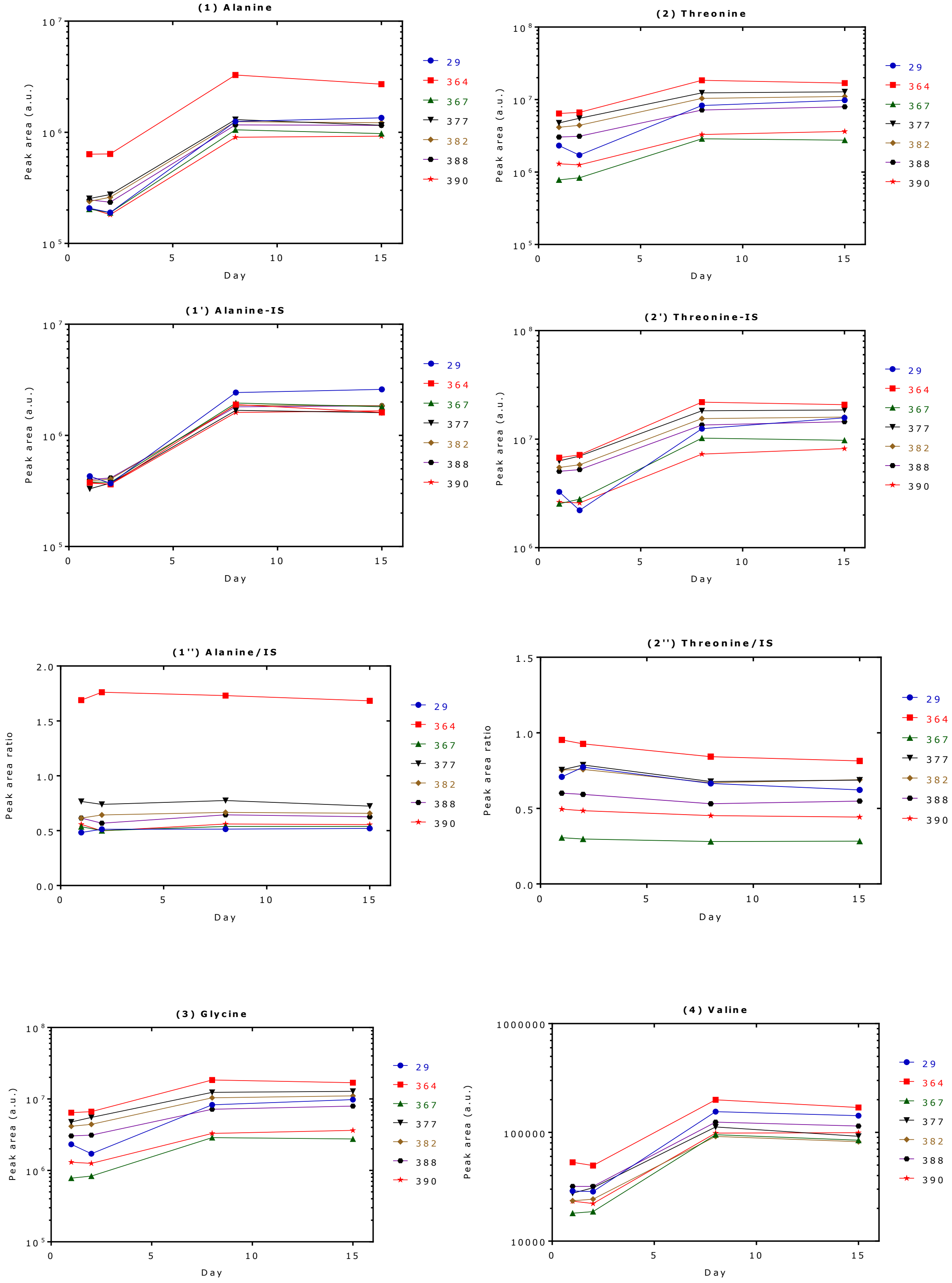

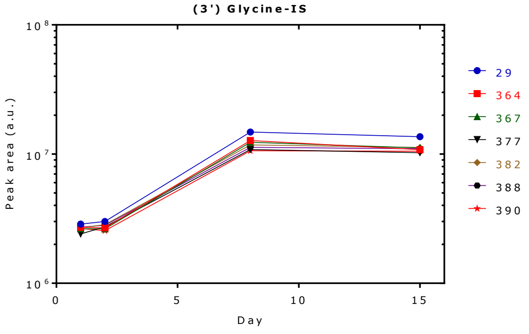
(3') Glycine-IS
Peak area (a.u.)
Day
29
364
367
377
382
388
390

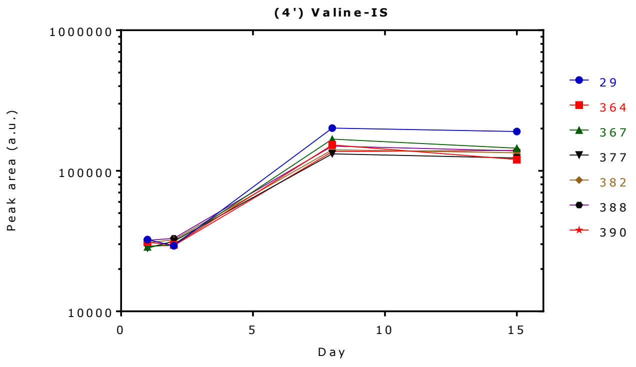
(4') Valine-IS
Peak area (a.u.)
Day
29
364
367
377
382
388
390

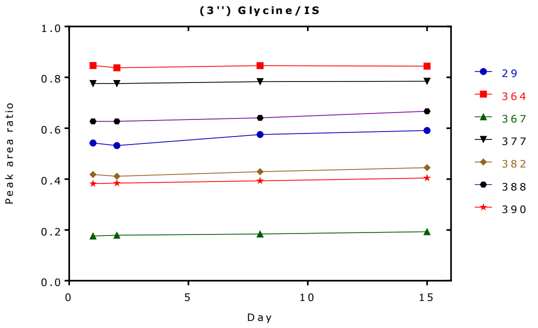
(3'') Glycine/IS
Peak area ratio
Day
29
364
367
377
382
388
390

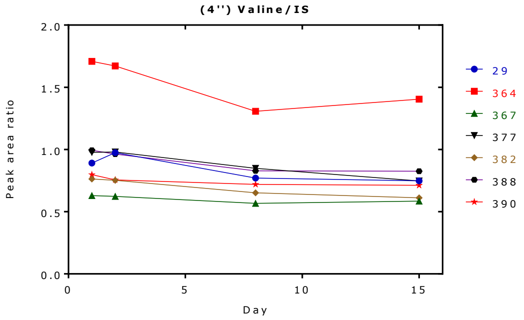
(4'') Valine/IS
Peak area ratio
Day
29
364
367
377
382
388
390

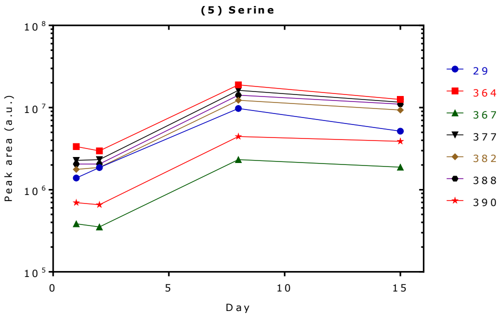
(5) Serine
Peak area (a.u.)
Day
29
364
367
377
382
388
390

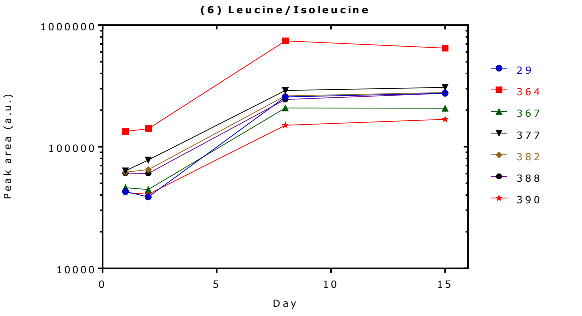
(6) Leucine/Isoleucine
Peak area (a.u.)
Day
29
364
367
377
382
388
390

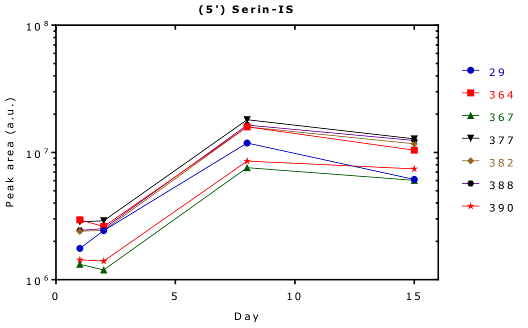
(5') Serin-IS
Peak area (a.u.)
Day
29
364
367
377
382
388
390

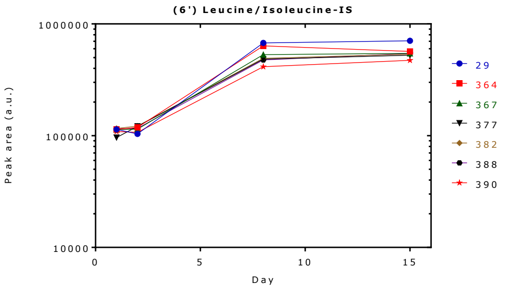
(6') Leucine/Isoleucine-IS
Peak area (a.u.)
Day
29
364
367
377
382
388
390

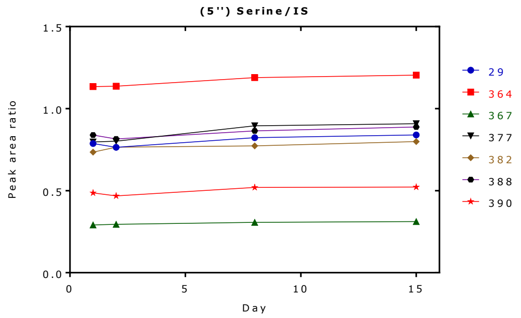
(5'') Serine/IS
Peak area ratio
1.5
1.0
0.5
0.0
0
5
10
15
Day
29
364
367
377
382
388
390

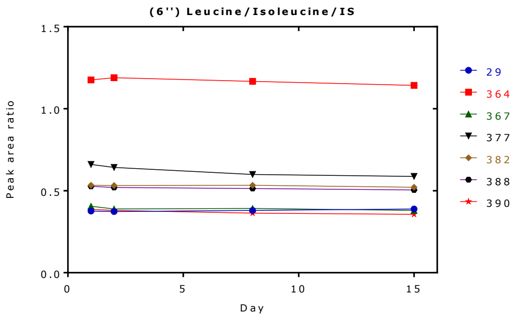
(6'') Leucine/Isoleucine/IS
Peak area ratio
1.5
1.0
0.5
0.0
0
5
10
15
Day
29
364
367
377
382
388
390

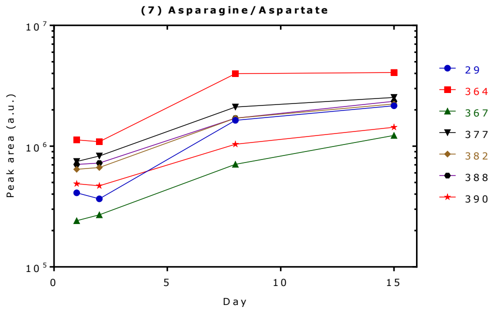
(7) Asparagine/Aspartate
Peak area (a.u.)
10^7
10^6
10^5
0
5
10
15
Day
29
364
367
377
382
388
390

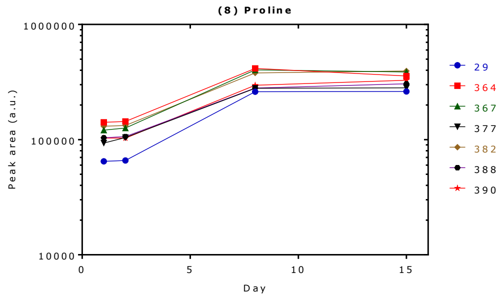
(8) Proline
Peak area (a.u.)
1000000
100000
10000
0
5
10
15
Day
29
364
367
377
382
388
390

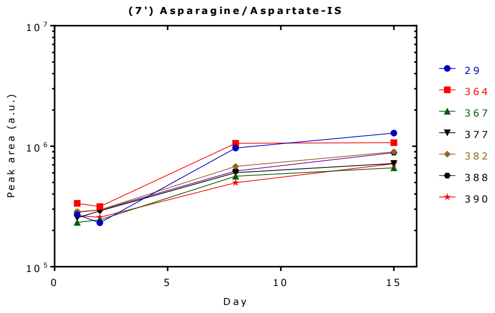
(7') Asparagine/Aspartate-IS
Peak area (a.u.)
10^7
10^6
10^5
0
5
10
15
Day
29
364
367
377
382
388
390

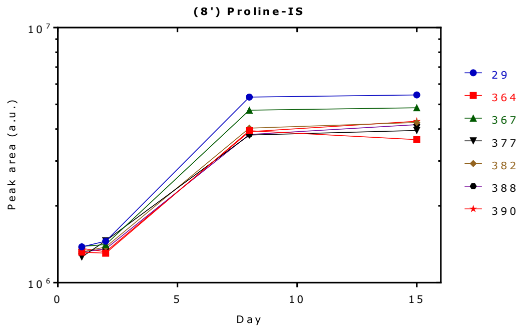
(8') Proline-IS
Peak area (a.u.)
10^7
10^6
0
5
10
15
Day
29
364
367
377
382
388
390

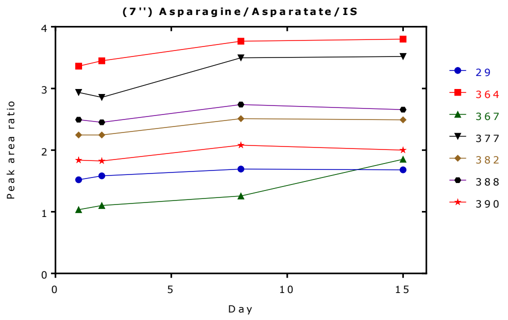
(7'') Asparagine/Asparatate/IS
Peak area ratio
4
3
2
1
0
0
5
10
15
Day
29
364
367
377
382
388
390

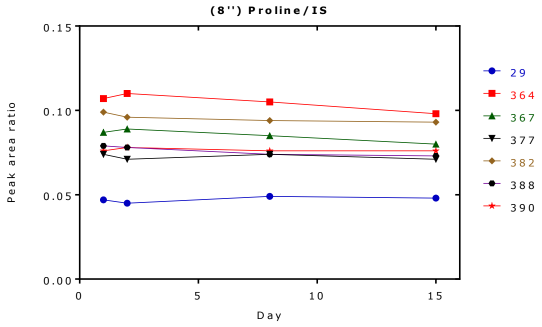
(8'') Proline/IS
Peak area ratio
0.15
0.10
0.05
0.00
0
5
10
15
Day
29
364
367
377
382
388
390

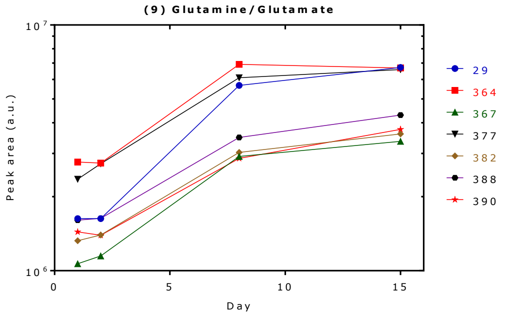
(9) Glutamine/Glutamate
Peak area (a.u.)
Day
10^7
10^6
0
5
10
15
29
364
367
377
382
388
390

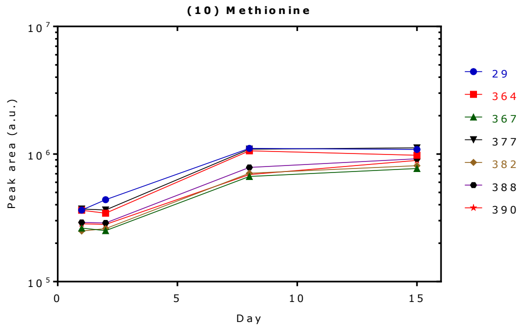
(10) Methionine
Peak area (a.u.)
Day
10^7
10^6
10^5
0
5
10
15
29
364
367
377
382
388
390

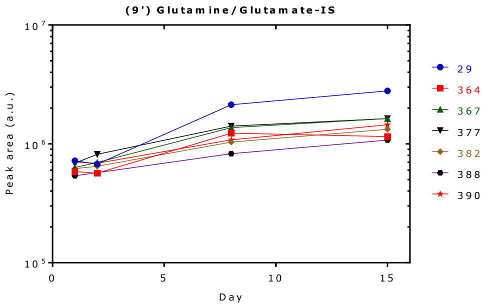
(9') Glutamine/Glutamate-IS
Peak area (a.u.)
Day
10^7
10^6
10^5
0
5
10
15
29
364
367
377
382
388
390

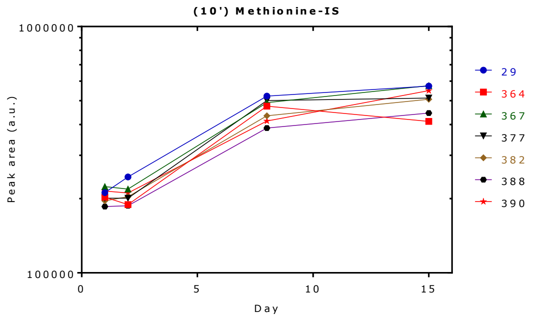
(10') Methionine-IS
Peak area (a.u.)
Day
1000000
100000
0
5
10
15
29
364
367
377
382
388
390

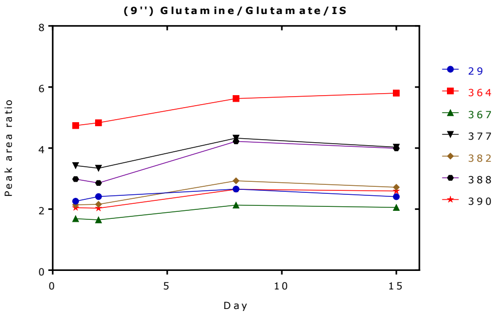
(9'') Glutamine/Glutamate/IS
Peak area ratio
Day
8
6
4
2
0
0
5
10
15
29
364
367
377
382
388
390

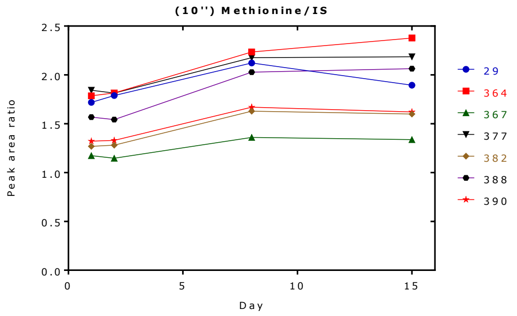
(10'') Methionine/IS
Peak area ratio
Day
2.5
2.0
1.5
1.0
0.5
0.0
0
5
10
15
29
364
367
377
382
388
390

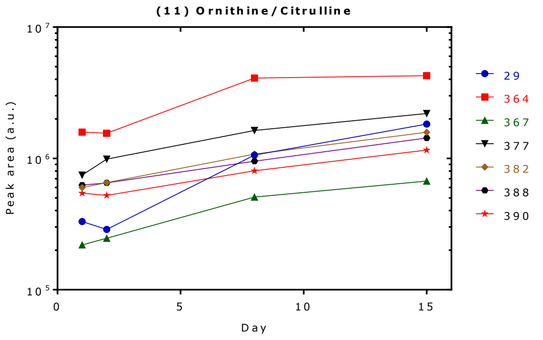
(11) Ornithine/Citrulline
Peak area (a.u.)
Day
10^7
10^6
10^5
0
5
10
15
29
364
367
377
382
388
390

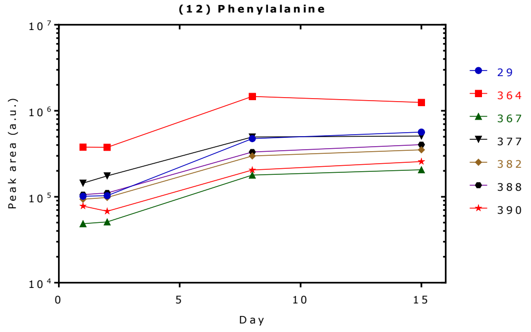
(12) Phenylalanine
Peak area (a.u.)
Day
10^7
10^6
10^5
10^4
0
5
10
15
29
364
367
377
382
388
390

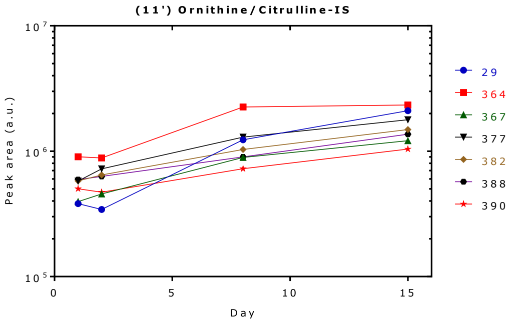
(11') Ornithine/Citrulline-IS
Peak area (a.u.)
Day
29
364
367
377
382
388
390

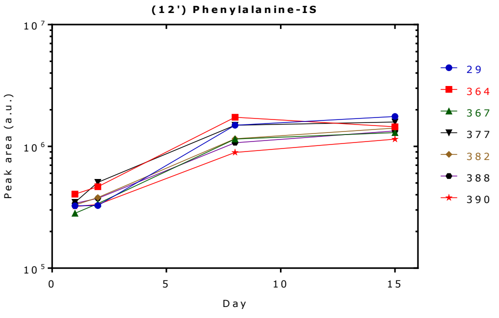
(12') Phenylalanine-IS
Peak area (a.u.)
Day
29
364
367
377
382
388
390

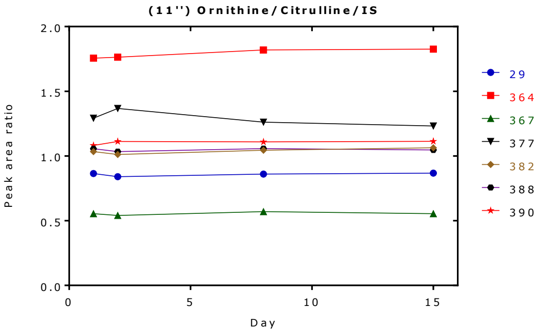
(11'') Ornithine/Citrulline/IS
Peak area ratio
Day
29
364
367
377
382
388
390

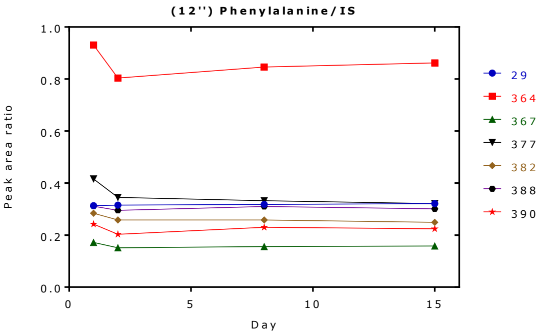
(12'') Phenylalanine/IS
Peak area ratio
Day
29
364
367
377
382
388
390

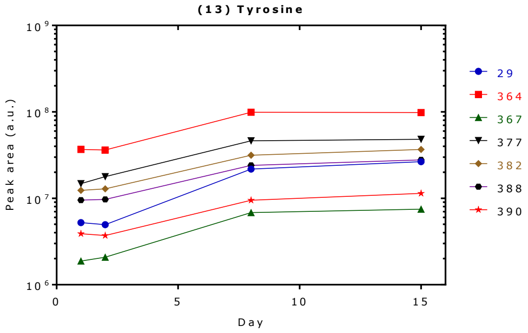
(13) Tyrosine
Peak area (a.u.)
Day
29
364
367
377
382
388
390

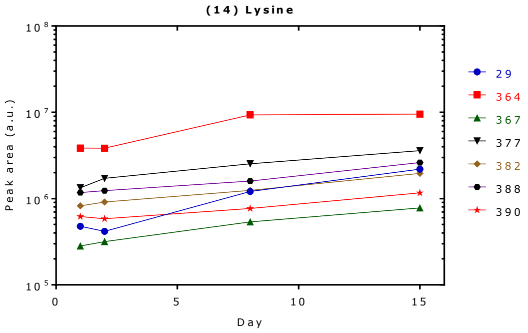
(14) Lysine
Peak area (a.u.)
Day
29
364
367
377
382
388
390

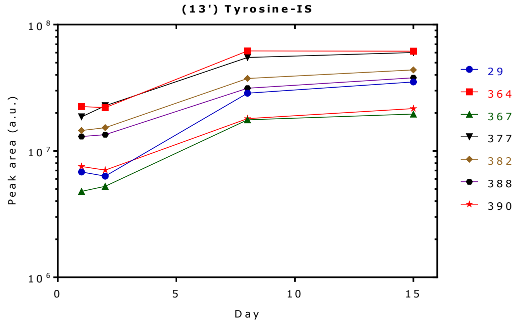
(13') Tyrosine-IS
Peak area (a.u.)
Day
29
364
367
377
382
388
390

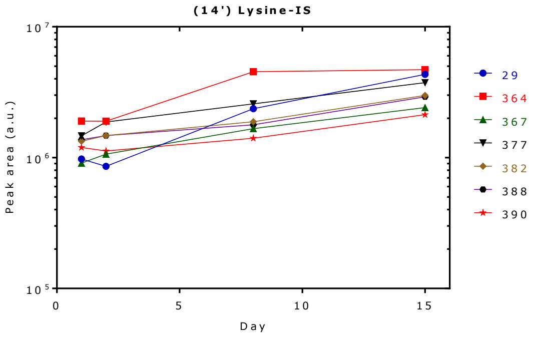
(14') Lysine-IS
Peak area (a.u.)
Day
29
364
367
377
382
388
390

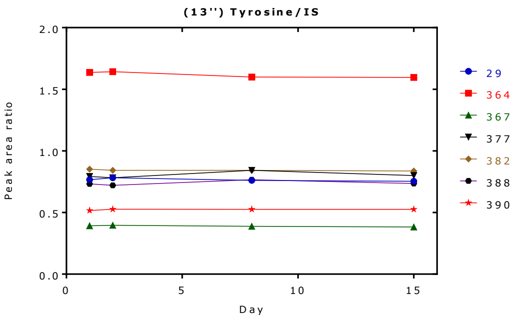
(13'') Tyrosine/IS
Peak area ratio
0.0
0.5
1.0
1.5
2.0
Day
0
5
10
15
29
364
367
377
382
388
390

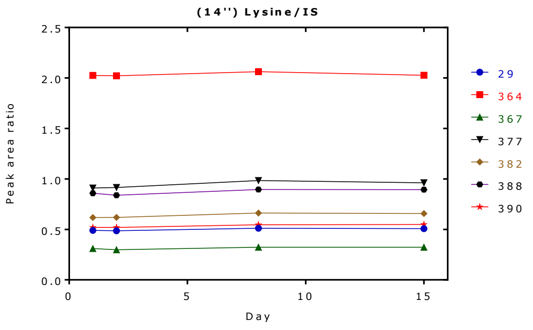
(14'') Lysine/IS
Peak area ratio
0.0
0.5
1.0
1.5
2.0
2.5
Day
0
5
10
15
29
364
367
377
382
388
390

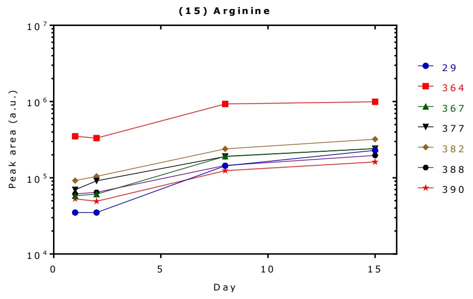
(15) Arginine
Peak area (a.u.)
10^4
10^5
10^6
10^7
Day
0
5
10
15
29
364
367
377
382
388
390

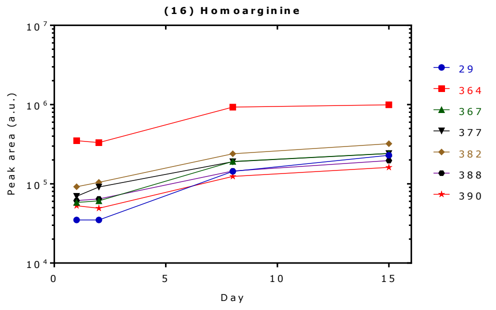
(16) Homoarginine
Peak area (a.u.)
10^4
10^5
10^6
10^7
Day
0
5
10
15
29
364
367
377
382
388
390

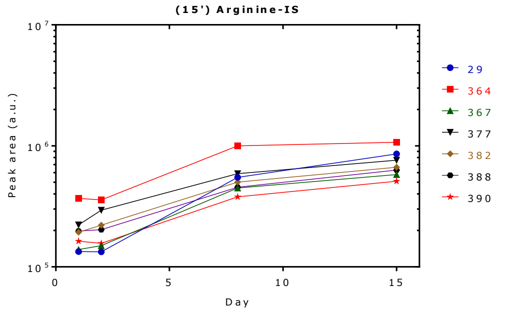
(15') Arginine-IS
Peak area (a.u.)
10^5
10^6
10^7
Day
0
5
10
15
29
364
367
377
382
388
390

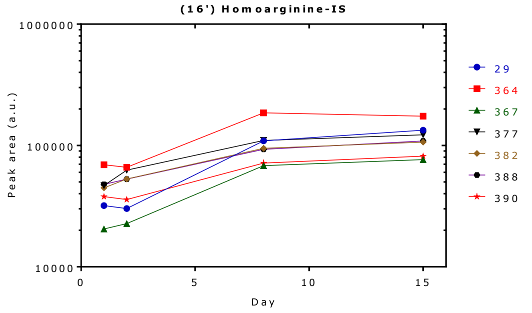
(16') Homoarginine-IS
Peak area (a.u.)
10000
100000
1000000
Day
0
5
10
15
29
364
367
377
382
388
390

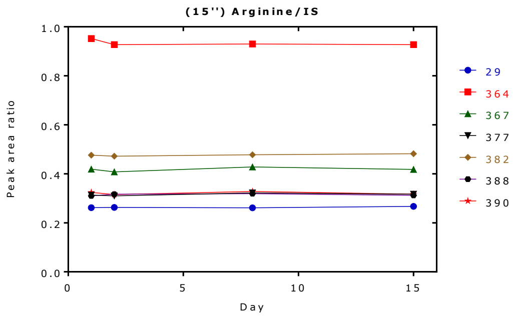
(15'') Arginine/IS
Peak area ratio
0.0
0.2
0.4
0.6
0.8
1.0
Day
0
5
10
15
29
364
367
377
382
388
390

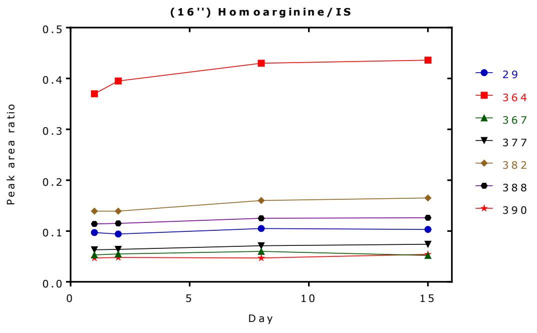
(16'') Homoarginine/IS
Peak area ratio
0.0
0.1
0.2
0.3
0.4
0.5
Day
0
5
10
15
29
364
367
377
382
388
390

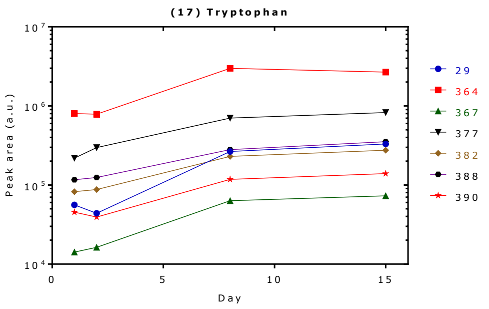

(17) Tryptophan
Peak area (a.u.)
Day
29
364
367
377
382
388
390

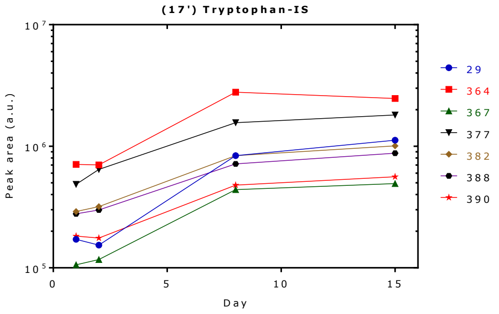

(17') Tryptophan-IS
Peak area (a.u.)
Day
29
364
367
377
382
388
390

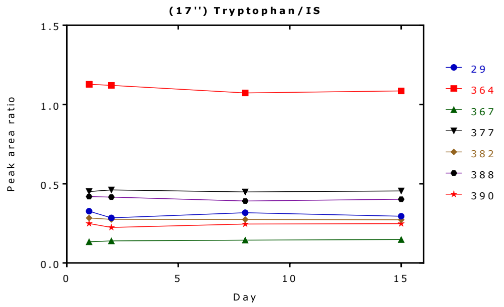

(17'') Tryptophan/IS
Peak area ratio
Day
29
364
367
377
382
388
390
